# Biography of Contemporary Physicians of Texas

**Published:** 1886-03

**Authors:** 


### BIOGRAPHY OF COTEMPORARY PHYSICIANS OF TEXAS.

WE are much pleased to learn through Messrs. Dixon & Netherly, the publishers who have charge of this work, who are now canvassing the State in its interest, that they are meeting with the utmost favor everywhere, and that three-fourths of the desirable material for the work is being secured. We publish below some of the names of physicians whose biography (some with and some without portrait) will appear in the work. As it is intended to make a thorough canvas and a complete success of the work, Mr. Dixon tells us that the appearance of the work will be somewhat delayed. However, the success of the work is assured. It will be illustrated with handsomely executed portraits of prominent physicians.

Those who desire to contribute their biographical data, from which the editor can write their biography to go in the collection, should correspond at once with Messrs. S. H. Dixon & Co.

The following is a partial list of those whose biography will appear in the work, in addition to those previously published; the last fifty have been very recently contributed. Those marked * have sent in their photograph. Time is slipping away. Don't neglect this matter any longer.

*Dr T J Heard, 1st President Texas State Medical Association,
* " Ashbell Smith, Ex " " " " "
* " R H Harrison, " " " " " "
" J H Pope, " " " " " "
* " S F Starly, " " " " " "
" A G Clopton, " " " " " "
* " H C Ghent, " " " " " "
* " H W Brown, " " " " " "
" D R Wallace, " " " " " "
* " A R Kilpatrick, " " " " " "
" E P Becton, President " " " "
" J D Osborn, State Medical Examiner Knights of Honor,
" R M Swearingen, State Health Officer,
* " W J Burt, Secretary Texas State Medical Association,

| | | |
|---|---|---|
| " S H Stout, | " J W Douglass, | " W M Monson, |
| * " G A Feris, | " G W Kerr, | " J M Morrison, |
| " T C Osborn, | " W G Jameson, | " A H Ketchum, |
| " S F Styles, | " Eug Clark, | " J A McQueen, |
| " L H Hardy, | * " R Menger, | " J S Lipscomb, |
| " O Morse, | " F R Martin, | " J S Holland, |
| * " J Cummings, | " A G Pendleton, | " J S Pugh, |
| * " M A Taylor, | " P C Woods, | " J P Barnett, |
| " W W Reeves, | " E Gazley, | " B S Glass, |
| " M D Sterrett, | " Jared Wilson, | " C C Black, |
| " R T Knox, | " J P Stephens, | " V H Reid, |
| * " S R Burroughs, | " T S Pettey, | " S E Carrington, |
| " Pow Jordan, | " S T Lowrey, | " I R Johnson, |
| * " J M Fort, | " E De Steiger, | " G M D Patterson |
| " E A Swepston, | " C Salms, | " I W Royston, |
| " W C Welch, | " Dan'l Lines, | " E N Shaw, |
| " H H Darr, | " J D Reid, | " J M Hons, |
| " M M Myers, | " J R Black, | " J A Fields, |
| " J K Mayo, | " W M Burger, | " W A Lockett, |
| " E J Beall, | " W W Urban, | " J Y Vermillion, |
| * " O Eastland, | " J H Morrison, | " C C Rutherford, |
| * " W T Baird, | " C W Legrand, | " J R Williamson, |
| * " W H Park, | " W P Fleming, | " Luc'n Campbell. |
| " W P Powell, | " D Monroe, | |

Others of the above have spoken to have their portraits inserted, but photos are not yet to hand.

